# Toward routine utilisation of native mass spectrometry as an enabler of contemporary drug development

**DOI:** 10.1039/d5md00617a

**Published:** 2025-07-30

**Authors:** Louise M. Sternicki, Sally-Ann Poulsen

**Affiliations:** a Institute for Biomedicine and Glycomics, Griffith University Gold Coast Queensland 4222 Australia s.poulsen@griffith.edu.au; b School of Environment and Science, Griffith University Brisbane Queensland 4111 Australia

## Abstract

As therapeutic modalities increasingly diversify, the need for biophysical tools for routine characterisation of the underlying biomolecular targets and their noncovalent interactions is growing. In this Opinion article we discuss the role of native mass spectrometry (nMS), a mass spectrometry technique where the intact biomolecule and its noncovalent interactions are preserved during the analysis, to gain important insights to guide drug discovery and development. We conclude that nMS is one of the most powerful technologies available with potential to rapidly advance multiple stages of therapeutic discovery and development, yet it is arguably underutilised. Specifically, we highlight how nMS may progress research for contemporary therapeutic modalities including those implicated in targeted protein degradation, fragment-based drug discovery and mRNA therapies.

## Abstract 2

We discuss how native mass spectrometry (nMS) is contributing important analytical insights to guide contemporary drug discovery and development. We focus on diverse modalities including targeted protein degradation (TPD), fragments (FBDD) and mRNA.

## Graphical abstract

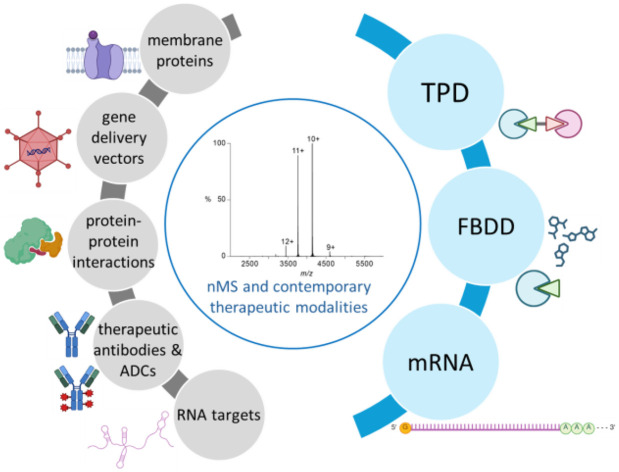

## Introduction

The development and implementation of enabling technologies in drug discovery is critical to provide advances that match the demands of an increasingly diverse landscape of therapeutic modalities. Native mass spectrometry (nMS) is a powerful biophysical technique that provides the mass of intact biomolecules in their native folded state including their noncovalent interactions with key binding partners.^1–5^ Building on decades of pioneering research to develop instruments and experimental methods, nMS has slowly but steadily transitioned from a niche and specialist MS technique to a method whereby today's trained MS practitioners are from diverse research backgrounds.^1,6–9^ Furthermore, the wider medicinal chemistry community now can perform high resolution and high accuracy mass measurements of diverse biomolecular systems using commercially available MS infrastructure commonly found within an organisation's core facility. Our group has a long-standing research interest in the application of nMS in the context of the ever-changing landscape of modern drug discovery, including with targeted protein degradation, fragment-based drug discovery and RNA-targeting. In this Opinion article our intention is to place a spotlight on the potential of nMS methods to address the analytical demands of discovery and development presented by modern therapeutic modalities, bridging small molecule approaches for modulating protein and oligonucleotide targets and larger biologics such as antibodies or mRNA.

**Fig. 1:**
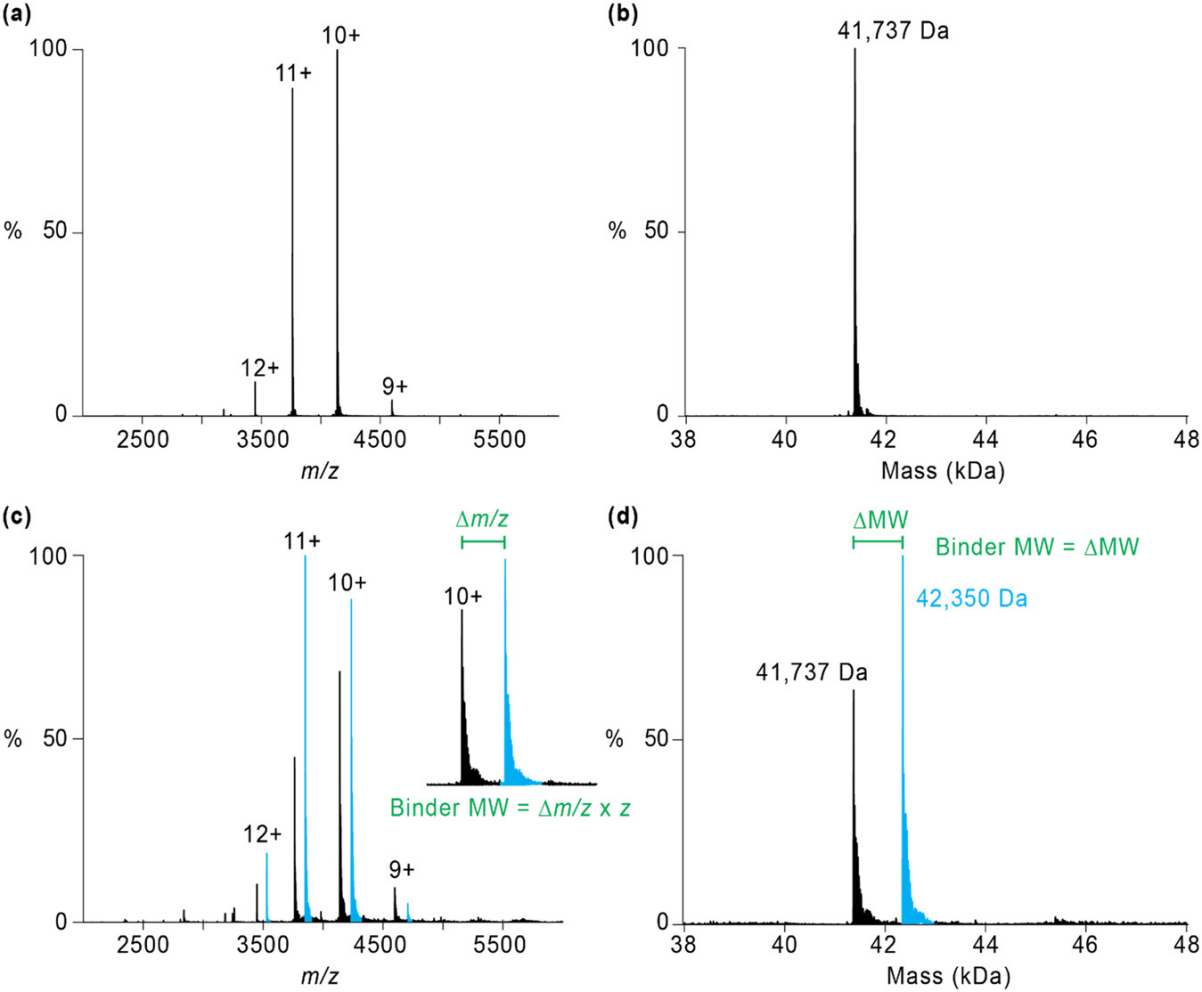
Schematic of raw (*x* axis is *m*/*z*) and deconvoluted (*x* axis is mass (kDa)) nMS data of a purified biomolecule sample without (a and b) or with an added interacting partner molecule or binder (c and d). The unbound biomolecule is the black trace; the bound biomolecule is the blue trace. Deconvolution provides the MW of the biomolecule species (bound and unbound) in the sample. Green annotations in (c and d) demonstrate the calculation of the MW (= identity) of the binder is possible using either raw or deconvoluted nMS data.

nMS can be considered gas-phase structural biology,^5,8,10^ with biomolecules and their noncovalent complexes analysed from volatile ‘physiological’ solution conditions (typically 100–200 mM ammonium acetate and neutral pH) that are ionised and transferred to the gas phase for detection. The nMS experimental readout is a mass spectrum comprising peaks corresponding to the sample components measured as multiply charged ions with a mass (*m*) to charge (*z*) ratio, *m*/*z*, providing an indirect measurement of mass. The *m*/*z* value is, however, straightforward to convert to molecular weight (MW) provided the observed signals can be uniquely mapped to a charge state *z* using a process termed deconvolution,^11^ with the MW affording the sample component's identity, Fig. 1.^12^ The broad scale of the *m*/*z* readout enables the multiple solution components (*i.e.*, multiple *m*/*z* values) to be readily distinguished and identified from a single mass spectrum. This ability of nMS to simultaneously detect all species present in each sample is critical for the applications discussed herein. Furthermore, nMS analysis of complex samples comprising noncovalent interactions can provide access to metrics including binding strength, stoichiometry, thermodynamic parameters and kinetics, in addition to other high-order structural information relevant to the interaction such as conformational changes, stoichiometries or oligomeric state.^1,7,11,13^ Advantages of the nMS technique to complement other methods for direct measurements of biomolecular interactions include speed and automation, low sample consumption, and label free measurements direct from a sample solution. These advantages cannot be understated, particularly when the biomolecules of interest can only be generated in limited amounts, are heterogeneous, not amenable to covalent modification or other labelling and/or not able to crystallise. Under such circumstances nMS can offer an unrivalled scope across the therapeutic landscape when compared to other structural biology techniques in use.

## Characterisation of biomolecules using native mass spectrometry

**Fig. 2:**
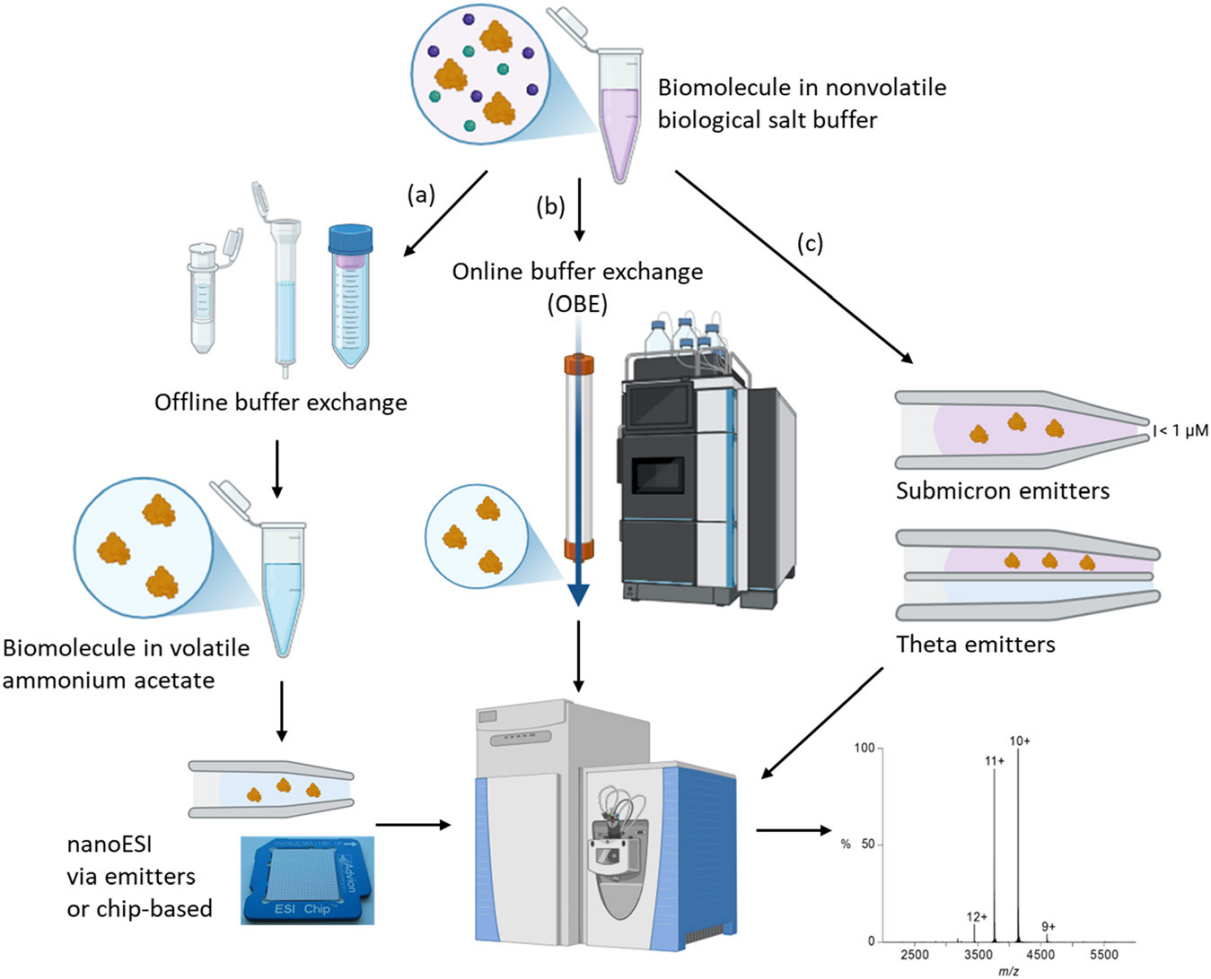
Alternative workflows for nMS analysis of biomolecules where samples are typically exchanged from their nonvolatile biological buffers into volatile ammonium acetate. Buffer exchange methods may be (a) manual offline buffer exchange methods (*e.g.*, centrifugal spin columns, gravity size exclusion or dialysis); or (b) semi-automated/automated online buffer exchange *via* size exclusion chromatography directly coupled to the MS; or (c) alternatively, altered emitters (submicron or theta emitters) have allowed nMS analysis of biomolecules directly from the nonvolatile storage buffer.

Biomolecular characterisation by nMS is typically performed using nanoelectrospray ionisation (nanoESI) where single-use small diameter spray capillaries or emitters are used to introduce the sample into the instrument in place of standard infusion ESI.^14–16^ NanoESI hardware enables reduced sample flow rates and smaller initial droplet sizes, this in turn enables gentler mass spectrometry instrument parameters for desolvation and ionisation and fewer charge states that collectively improve the preservation of weak noncovalent interactions.^14–16^ The use of nanoESI nMS can make possible the production of mass spectra using only a few picomoles of native biomolecule, vital when working with biochemical species which are difficult to produce in the quantities required for standard ESI or clinical samples with limited, non-replenishable supply. Importantly, nanoESI provides better sample tolerance to nonvolatile salts that are common in biomolecule samples and overall increased sensitivity of measurements. Even so, biomolecule analysis with nMS usually requires ‘buffer exchange’ of the sample into a volatile solution that is compatible with nMS prior to analysis, a time-consuming step when performed offline (manually) that negates the benefits of automation of other steps of the nMS analysis and also limits nMS applications with less stable biomolecules, Fig. 2(a).^17^ This drawback has been addressed with automated and rapid (<5 minute) online buffer exchange (OBE) directly coupled with nMS, demonstrating an avenue for high sample throughput^18^ and also improved compatibility for low stability samples as they are out of their preferred non-volatile storage buffer for a shorter period of time prior to nMS analysis, Fig. 2(b).

Another approach circumventing the requirement for tedious manual buffer exchange is the use of submicron emitters to introduce the biological sample into the MS instrument, where the even smaller diameter openings (nm diameters) improve tolerance to salt, desalting and desolvation, such that it makes possible the analysis of biomolecule samples direct from high salt biological buffers (*e.g.*, Tris, HEPES, NaCl) without buffer exchange, Fig. 2(c).^19^ More, recently this has also been demonstrated using theta emitters that comprise two internal channels, with the analyte in biological buffer in one channel and ammonium acetate solution in the second channel, and mixing of the samples occurring at the point of nanoESI, Fig. 2(c).^20^ While the use of modified (theta or submicron) emitters is yet to be demonstrated with a broad range of analytes, with the emitters typically produced in-house and workflows low in throughput, they may in future help to expand access of nMS analysis to biomolecules including those not stable in traditional volatile nMS buffers. Automation of nMS using chip-based nanoESI (Advion Nanomate)^21^ has dramatically increased throughput to improve the speed of applications to biomolecular screening, while the chip design comprises individual nozzles that eliminate carryover effects between samples, a further advantage when high quality data is sought.^6,21^

One of the core parameters that nMS can inform that is of relevance in drug discovery is binding affinity (*K*_D_) as both the unbound and bound species in a sample can be measured simultaneously. The *K*_D_ may be determined using a traditional titration approach or alternatively a more rapid single concentration approach. The choice of which approach to deploy may depend on the level of detail required from the measurement, with rapid triaging of a screening library possible with the single concentration *K*_D_ method and a more in-depth characterisation of interactions possible with a titration *K*_D_. For the single concentration approach to give meaningful *K*_D_ values it needs the biomolecule and its binding partner to be at relevant concentrations respective to their binding affinity, with the use of control systems of known *K*_D_ values best practice to validate the method.^7^ These quantitative approaches were recently reviewed in full, and we direct interested readers to this review.^7^

**Fig. 3:**
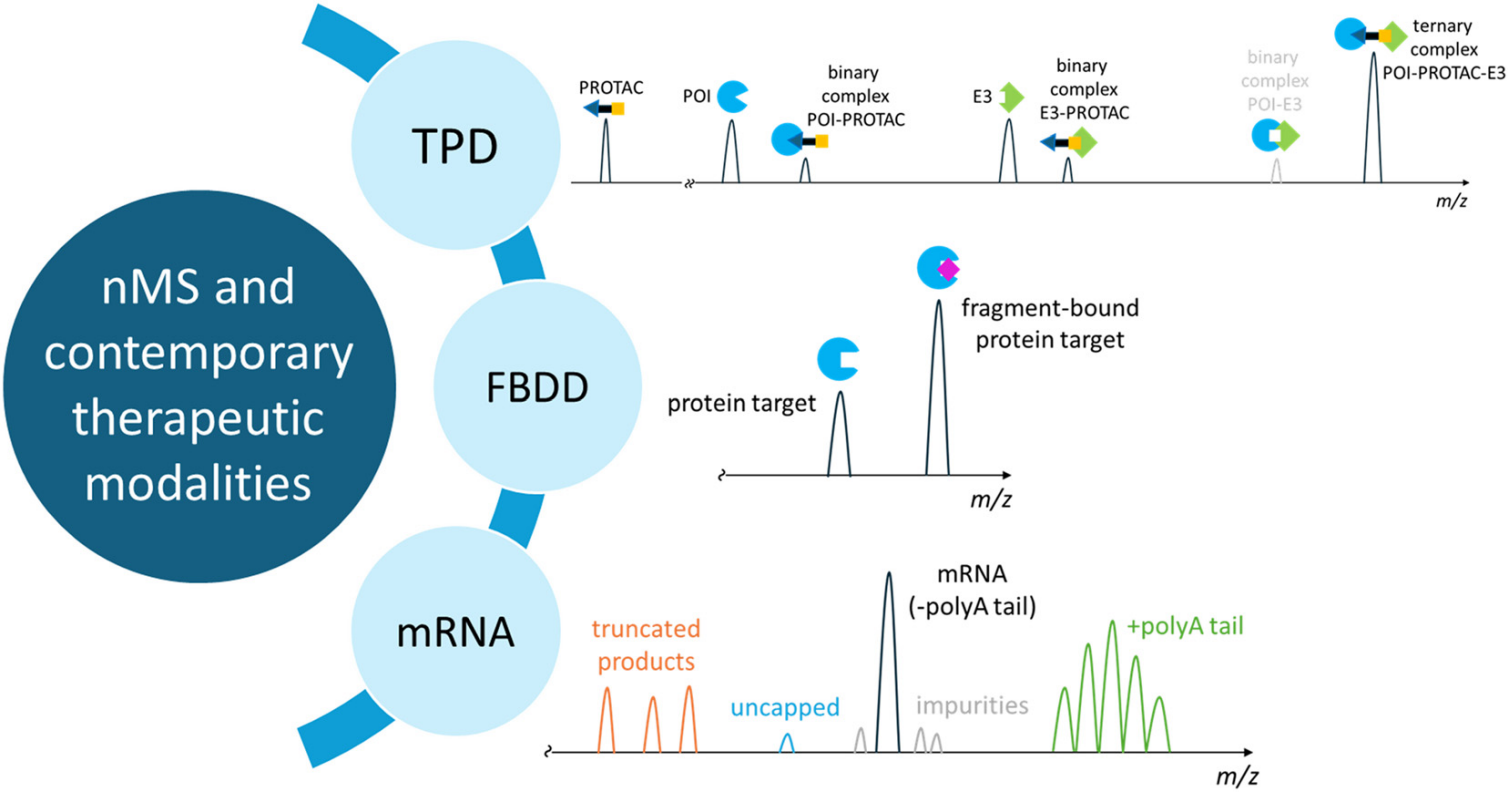
Application of native mass spectrometry (nMS) with contemporary therapeutic modalities of targeted protein degradation (TPD), fragment-based drug discovery FBDD and mRNA. PROTAC: PROteolysis TArgeting Chimera; POI: protein of interest; E3: E3 ubiquitin ligase.

Advances in instrument hardware^2,6^ and charge detection mass spectrometry^22–24^ now enable characterisation of biomolecules from heterogenous populations with essentially unrestricted upper size limits. Using commercial instruments, megadalton protein assemblies up to 18 MDa have been successfully characterised^2^ with mass analysis of intact mRNA analysis recently reaching ∼3 MDa.^25^ These large and heterogeneous systems still predominantly remain in the realms of mass spectrometry specialists, with advanced operational experience and expertise needed to unravel the information present within the spectral complexity. An important consideration for wider use is a need to establish and harmonise fundamental methods for these sample types as a safeguard against misinterpretation of complex spectra that could lead to discrepancies or errors entering and contaminating the scientific literature.^11^ In this Opinion we will focus on application of nMS to three contemporary therapeutic modalities, targeted protein degradation, fragment-based drug discovery and mRNA therapies. These are reflective of drug discovery approaches where nMS is not used extensively but we anticipate that the benefits of nMS approaches can have a major impact in the future to complement other more common biophysical approaches and further advance progress towards new medicines, Fig. 3.

### nMS and targeted protein degradation

Targeted protein degradation is one of the most compelling contemporary approaches for therapeutic drug discovery against proteins that drive disease, with >25 degrader drug candidates currently in clinical trials.^26,27^ There are two prominent subclasses of degraders: PROteolysis TArgeting Chimeras (PROTACs) and molecular glues (MGs), both are small molecules that hijack the endogenous ubiquitin–proteasome system to induce ubiquitination of a target protein, marking it for degradation by the proteasome. This mechanism of action leads to a longer duration of action than classical small molecule inhibitors as well as the ability to target and degrade previously considered undruggable proteins, hence, the attractiveness and strong interest in this method.^26^

PROTACs comprise two distinct parts, one that recruits the target protein (also called the neo-substrate) and a second that recruits an E3 ligase, with the two parts covalently linked. MGs comprise a single pharmacophore that directly recruits and binds to both the target protein and the E3 ligase. The formation of the ternary complex comprising the E3 ligase, degrader molecule and protein target, is the driver for productive target degradation. Characterising this critical interaction is immensely important for degrader discovery and development, yet there is a paucity of direct, sensitive, systematic and quantitative methods to do so, with a reliance on protein crystallisation or stringent sample preparation for X-ray crystallography/cryo-electron microscopy, or protein modification needed for alternative biophysical methods (*e.g.*, SPR) or cell-based techniques (*e.g.*, NanoLuciferase and HaloTag reporters). This presents a significant barrier for those working to develop degraders as there is a vast number of proteins of interest that may be too flexible or too dynamic to crystallise or that are not amenable to modification as it interferes with function or structure. Recently we reviewed the reported contributions of nMS to targeted protein degradation,^9^ while small in number the samples comprised three different PROTACs^28–30^ and seven different MGs^31,32^ using proteins expressed from *E. coli* or SF9 insect cells. The nMS data was acquired on a range of different commercially available instruments, with samples in 100–200 mM ammonium acetate and measured ternary complexes ranging in size from ∼57 kDa to 180 kDa. Since our 2023 review (also the first review on this topic), we have identified several new studies that use nMS to characterise PROTAC or MG mediated ternary complexes.^33–36^ There are also two additional reported reviews on the topic,^37,38^ possibly a reflection of the important value of the emerging contribution of nMS with targeted protein degradation.

We anticipate that as more E3 ligases are discovered (so far only a handful of the >600 E3 ligases are in use with targeted protein degradation) and where the samples associated are more complex and more challenging for ternary complexes to be characterised by X-ray crystallography or cryo-electron microscopy, that nMS will become invaluable as an alternative structural biology method. We also foresee that because nMS could study more ternary complexes with different ligands more routinely that it could play an important role to triage the best candidate ligands to advance to X-ray crystallography or cryo-electron microscopy analysis where comprehensive ligand screening is not viable (*e.g.*, sample quantity, time or cost are prohibitive), hence, providing a substantial platform for the field to advance. Noting the first PROTAC ternary complex crystal structure was only published in 2017,^39^ this is particularly an avenue where nMS has potential to generate protein ternary complex interaction data at scale to fast-track future degrader development. Specifically, nMS could assist with the stoichiometry and identity of complex constituents and quantifying binding (*K*_D_s) using a combination of established commercial software or opensource software.

Native charge detection mass spectrometry (nCDMS) where the masses of individual ions are determined by simultaneous measurement of their mass-to-charge ratio (*m*/*z*) and charge (*z*)^40–42^ may also facilitate production of high-resolution spectra to accurately characterise high mass degrader complexes. With CDMS the amplitude of the image current generated by each individual ion corresponds to peak intensity and makes possible a measure of ion charge (*z*). With both *m*/*z* and *z* available for all measured ions accurate mass distributions can be determined for heterogeneous and/or high molecular weight species that are typically not amenable for meaningful analysis with standard nMS.^40–42^ The caveat with this is the same as for other biophysical methods, trained users with a strong understanding of the underlying principles of the method are integral for the contribution of quality data to support research endeavours. Although other proximity inducing agents such as protein–protein interaction stabilisers are not discussed here, nMS has been used to characterise ternary complexes of these agents,^43^ and it stands to reason that nMS could provide opportunities to accelerate broadly the development of reagents that act *via* a proximity associated mechanism.

### nMS and fragment-based drug discovery

Fragment based drug discovery (FBDD) is another therapeutic approach where bridging the divide between small molecule libraries and their downstream therapeutic development can be enhanced by adopting nMS. An underlying principle of FBDD is that fragments of interest bind weakly but optimally to their target, with weak binding necessitating sensitive biophysical techniques to identify hits from non-binding fragments.^44,45^ Fragment hits are commonly identified using biophysical methods to screen curated fragment libraries, either commercially available or commercial-in-confidence libraries where the underlying library construction is of intrinsic value.^46,47^ A key advantage of fragment screening is the efficient coverage of chemical space that fragments provide due to their small molecular size, with complete screening campaigns possible using substantially smaller (by number) compound libraries than is typically needed for success from high throughput screening (HTS) libraries made up of larger mass compounds.^48^

We first reviewed the contributions of nMS to guiding fragment screening for hit identification in 2013 (ref. 49) and subsequently published an update in 2023.^50^ These reviews provide both a comprehensive perspective on the early^51–54^ and then later adoption of nMS for fragment screening.^55–59^ We concluded that nMS is particularly well suited for supporting fragment screening campaigns by allowing the pivotal detection of weakly binding ligands (binding constants as low as mM), and nMS even supports screening of pooled fragments or multiplexed proteins. Despite the advantages, nMS is not used to the extent of the more common biophysical methods, including those where there may be significant challenges such as X-ray crystallography, that requires proteins suitable for crystallisation, or NMR, where proteins are commonly isotopically labelled and high fragment stock concentrations are needed, that can frequently meet with insufficient solubility leaving many fragments unsuitable. The trend in use of different biophysical methods for fragment screening has been recorded by informal polls of the Practical Fragments Blog community in 2011, 2013, 2016, 2019 and 2024.^60^ The most common methods are consistently X-ray crystallography, NMR and SPR, but MS approaches (comprising all MS-based approaches, not just nMS) have risen notably from <10% of users in 2011 to >25% in 2024. At the time of writing there are seven approved fragment-derived drugs and more than 50 fragment-derived compounds in various stages of clinical development,^47,61–63^ with nMS not yet playing a substantial role in those examples, but a situation we expect will change for future FBDD-derived drugs.

Recently, the use of covalent fragments for FBDD has emerged,^64,65^ with covalent binding altering the technical considerations for biophysical screening requirements as compared to noncovalent fragment binding. Our group recently developed a nMS workflow for electrophilic fragment screening of pooled fragments.^66^ The screening method also enabled identification of the modified protein residue by utilising mutant proteins and supported direct simultaneous observation of orthosteric (noncovalent) and covalent fragment binding, not possible with denaturing MS methods. This powerful capability of nMS could greatly accelerate discovery of covalent drug discovery where there is a genuine need for screening technologies to characterise concurrent binding and support better understanding of covalent binding.

### nMS and mRNA as biotherapeutics

Despite the fast-moving landscape of mRNA development, product quality specifications (identity, quantity and purity) are not established for mRNA therapeutics. This is a significant issue as there is an expectation of consistent quality control as has been in place (and is expected) for alternative biologics such as protein-based therapeutics. The current draft guidelines released by the United States Pharmacopeia, August 2024 ‘Analytical Procedures for Quality of mRNA vaccines and therapeutics, 3rd edition’^67^ and the European Medicines Agency, March 2025 ‘Guideline on the quality aspects of mRNA vaccines’^68^ identify a pressing need for higher resolution analytical approaches that can monitor the integrity of the whole ‘intact’ mRNA. The challenge for implementing mRNA quality specifications is partly owing to technical and infrastructure considerations that are substantially different to those needed for protein-based biotherapeutics. The unique critical quality attributes (CQAs) of the mRNA therapeutic include the 5′-cap, the 3′-poly(A) tail length and heterogeneity, nucleotide modifications, and the overall mRNA identity and integrity. These CQAs significantly impact the mRNA stability, translational efficiency and efficacy. Furthermore, all mRNA-based vaccines on the market or in clinical trials are manufactured using *in vitro* transcription (IVT), with both shorter and extended RNA byproducts formed in the IVT reaction. These byproducts equate to impurities, and they can adversely impact mRNA production costs, efficacy and safety. To provide insight on the CQAs, as well as the identity and quantity of mRNA impurities (either during development, production or in the final product), current analytical methods must be improved or novel analytical tools introduced.^67,68^

To date there are only a few published examples of mRNA analysis by nMS.^25,69^ Genentech described the use of nMS to measure a 683 nt IVT mRNA and the heterogeneity that arose from the addition of a 3′ poly(A) tail. Analysis of the mRNA without the 3′-poly(A) tail (mRNA-) revealed a single product with a mass of 224 080 kDa.^69^ Addition of the 3′-poly(A) tail resulted in a 783 nt mRNA (mRNA+), which nMS confirmed had the expected mass difference that corresponded with the addition of the polyA tail. nMS of the mRNA+ also displayed higher heterogeneity due to the partially resolved variability in the number of adenosines constituting the 3′-poly(A) tail. Analysis of both species revealed MWs 3–4 kDa higher than expected due to the presence of noncovalently bound nucleotide fragments (*i.e.*, aborted transcripts), while in the mRNA+ sample a small number of minor variants 2.5 kDa larger were also observed, which corresponded to a small number of additional nucleotides (<10) and were hypothesised to be small dsRNA 3′-loop back byproducts. nMS analysis (at isotopic resolution) of the products from cleavage of the 3′-poly(A) tail by T1 RNase revealed a distribution from 95 to 110 adenosine residues. The approach of RNAse cleavage prior to nMS analysis simplified analysis (lower mass, reduced heterogeneity) and facilitated a more detailed assessment of the polyA tail modification compared to intact analysis of the full-length mRNA.

In an academic-industry collaboration Heck and colleagues together with Pfizer characterised intact mRNA-based therapeutics without digestion using both nMS (mRNA <1 MDa) and charge detection mass spectrometry (CDMS; mRNA >1 MDa).^25^ This study was inspired by the need for strategies specific for mRNA analytical challenges and the corresponding very limited tools available to characterise therapeutic mRNA.^70^ Through analysis of a panel of different mRNAs (ranging from 858 to 9400 nucleotides (283 kDa–3 MDa)) they demonstrated that nMS can reliably characterise the mass of mRNAs up to 1000 nucleotides, although this is considered mid-size mRNA. CDMS was used to access accurate mass measurements for higher mass mRNAs. For this an organic co-solvent was required to denature the RNA and give higher charged state species for improved ion behaviour, increased signal-to-noise and reduced charge uncertainty, leading to an overall increase in mass accuracy. They highlight the challenges for intact RNA analysis, that although shared with protein samples, is exacerbated by the inherent heterogeneity of mRNA as well as higher propensity of salt adducts (interferents of nMS) as a consequence of the negatively charged RNA backbone.^25^ An important take home message commented on in this study was ‘it should be noted that it is often difficult to find a fit-for-all MS method that would allow for optimal transmission and desolvation of all species from low-molecular-weight-species to high-molecular-weight species’, a reminder how biophysical methods used in combination and *via* collaboration are a way forward to strengthen drug discovery.

## Outlook and broader application of nMS in other contemporary drug discovery

The nMS applications covered in this Opinion were selected to put a spotlight on nMS as it relates to contemporary drug development. That said, we believe that the coverage we provided is the tip of the iceberg, and that nMS is genuinely underutilised in drug discovery and development. We wish to acknowledge other pioneering efforts of researchers in industry and academia advancing drug discovery for other challenging systems including membrane proteins,^71–76^ transient protein–protein interactions,^77–82^ therapeutic antibodies^83–86^ and gene delivery vectors.^87–89^ We recognise that long standing challenges remain for wider uptake of nMS in drug discovery – these are both technical (*e.g.*, affordable, commercially available and user-friendly instruments) and nontechnical (policies and environments that support research and research training across disciplines). As nMS may be used in concert with a vast array of allied methods (*e.g.*, ion mobility and collision-induced unfolding, hydrogen–deuterium exchange, collision dissociation, top-down sequencing) and methods in development (*e.g.*, soft landing nMS whereby analysed molecules are gently landed and collected post-MS analysis for further investigation, most recently optimised for cryo-electron microscopy structural characterisation)^90–94^ there is the promise of even greater advanced capability than we have discussed, for example with membrane proteins, the emergence of RNA targeting therapies, antibodies and antibody–drug conjugates, virus like particles and many other biomolecules. Additionally, recent advances in nMS are moving from purified biomolecules characterised in isolation, to endogenous biomolecules analysed in semi-purified or entirely native environments at their natural abundances and with their natural proteoforms and/or post-translational modifications, facilitating improved maintenance of relevant biomolecular structure and function, an especially important consideration for membrane proteins.^78,95–101^

We expect to witness advanced nMS becoming more common practice instead of residing predominantly in specialist laboratories using bespoke in-house modified instruments. We caution that as more researchers affiliated with drug discovery adopt nMS, that the community that has pioneered nMS will be presented with a greater need to establish standardised practices for the various steps of nMS analysis and continue to address the challenges of throughput, data reproducibility, data analysis and sharing so that it is fit-for-purpose in the fast pace of drug discovery and development settings. We hope to see training of tomorrow's nMS professionals so that they can provide the human element to critical nMS infrastructure, for example engaged in research directly or providing access to expertise in core analytical facilities in academia and industry, ultimately increasing the impact of nMS. Despite challenges, the applications of nMS in drug discovery and development has progressed markedly in recent decades, and with so much to offer for biomolecular analysis the future appears very bright indeed.

## Conflicts of interest

There are no conflicts to declare.

## Data Availability

As an Opinion there are no new data generated or shared.
